# Assessment of Predictive Factors That Shorten Duration of Treatment in Patients With Multiple Myeloma Using AI: Real-World Longitudinal Study Using Data From Medical Data Vision Claims Database

**DOI:** 10.2196/75586

**Published:** 2026-02-19

**Authors:** Hiroshi Handa, Tadao Ishida, Shuji Ozaki, Shinsuke Iida, Kittima Wattanakamolkul, Chika Sakai, Kenichi Kato, David Bin-Chia Wu, DaeYoung Yu, Shota Nemoto, Yasuho Yamashita, Takuma Shibahara

**Affiliations:** 1 Department of Hematology, Gunma University Hospital Gunma Japan; 2 Department of Hematology, Japanese Red Cross Medical Center Tokyo Japan; 3 Department of Hematology, Tokushima Prefectural Central Hospital Tokushima Japan; 4 Department of Hematology and Oncology, Nagoya City University Graduate School of Medical Sciences Nagoya Japan; 5 Value, Evidence and Access Department, Integrated Market Access Johnson & Johnson Tokyo Japan; 6 Medical Affairs Johnson & Johnson Tokyo Japan; 7 Asia Pacific Regional Market Access Johnson & Johnson Singapore Singapore; 8 Saw Swee Hock School of Public Health, National University of Singapore Singapore Singapore; 9 School of Pharmacy, Faculty of Health & Medical Sciences, Taylor’s University Malaysia Malaysia Malaysia; 10 Industrial & Digital Business Unit Hitachi (Japan) Tokyo Japan; 11 Research and Development Group Hitachi (Japan) Tokyo Japan

**Keywords:** duration of therapy, machine learning, artificial intelligence, multiple myeloma, prediction probability, database, Japan

## Abstract

**Background:**

With the availability of newer therapies, the duration of therapy (DoT) shortens with each increasing line of treatment in Japanese patients with multiple myeloma (MM).

**Objective:**

This study aimed to identify factors that shorten DoT in patients with MM using a machine learning (ML) procedure from the Medical Data Vision (MDV) database.

**Methods:**

This nationwide, retrospective observational real-world study was conducted using anonymized patient data from the MDV claims database from 2003 to 2022. Patients (≥18 y) with transplant-ineligible newly diagnosed MM (continued first line therapy), or relapsed or refractory MM (continued second or third line therapies) were included. To identify important predictive factors, an explainable deep learning model was created using 647 extracted variables (continuous, binary, and nominal categorical) from the MDV database, and the extracted data were used to train ML algorithms to build point-wise linear (PWL) models for predicting DoT. Predictive performance of the PWL model was compared with the elastic net (regularized logistic regression) and the extreme gradient boosting models, and calculated by area under the curve and evaluated by 10-fold double cross-validation. A clustering analysis (k-means method) of 4848 individual samples assessed the relationship between each sample and DoT (3, 6, and 12 months). The characteristics of clusters and sample features belonging to each cluster during and after treatment were studied.

**Results:**

Overall, 2762 (4848 individual samples) patients were evaluated (mean age 69.6, SD 11.8 years; 1450/2762, 52.5% male). The area under the curve score of the PWL model to predict DoT at 3, 6, and 12 months was 0.61, 0.64, and 0.66, respectively. Based on the similarity of coefficients of regression models, samples were categorized into 2 clusters (clusters A and B) at DoT of 3 months, 3 clusters (clusters A, B, and C) at 6 months, and 12 months (clusters A, B, and C). Cluster B versus cluster A (at 3 months) and cluster C versus cluster A and B (at 6 and 12 months) had a significantly (*P*<.01) higher pretreatment Charlson Comorbidity Index. They also showed a lower median of prediction probability. At 3 months in cluster B and at 6 and 12 months in cluster C, the use of immunomodulatory drugs for MM treatment was significantly higher in patients who met predicted DoT at each threshold versus those who did not. Additionally, the use of aspirin was significantly higher in cluster B and cluster C at 3 and 6 months, respectively.

**Conclusions:**

Applying ML techniques using the PWL model yielded efficient results to understand trends associated with treatment and characteristics of Japanese patients with MM whose DoT were shortened. The study demonstrated that patients’ disease status and management-related factors, including use of immunomodulatory drugs and management of thromboprophylaxis, may be associated with DoT length.

## Introduction

Multiple myeloma (MM), with an estimated annual incidence of 7.1 per 100,000 population, is the second most common hematologic neoplasm worldwide [1]. As per the Global Cancer Observatory report, there were 6988 new cases of MM and 4827 deaths in 2022 in Japan [2]. With a rapidly aging population, the incidence of MM is projected to increase [3-5].

The treatment choice for patients with newly diagnosed multiple myeloma (NDMM) is based on patient-based factors (age, frailty status, comorbidities, compliance, and lifestyle preference), disease-based factors (cytogenetics abnormalities, mutations, biochemical abnormalities, extramedullary disease, and International Staging System score), treatment-related factors (access to therapy, cost, and safety), and the eligibility to undergo autologous stem cell transplantation postinduction therapy [6]. In Japan, several key therapies have been approved for MM treatment over the past 2 decades: bortezomib (approved in 2006) [7], lenalidomide (approved in 2010) [8], pomalidomide (approved in 2014) [9], and thalidomide (approved in 2008) [10]. These agents have become standard treatment options, demonstrating clinically meaningful improvements in both progression-free and overall survival for patients with MM. For transplant-ineligible patients with NDMM and relapsed or refractory multiple myeloma (RRMM), novel therapies (such as panobinostat, elotuzumab, carfilzomib, ixazomib, daratumumab, and isatuximab) are commonly used treatment modalities [11,12].

Although patient survival is increasing with the advancement in treatment options and supportive care [4,5], MM is still considered an incurable disease as patient survival solely depends on the prescribed treatment regimen and the patient’s demographic and clinical characteristics [1].

Treatment continuation for an appropriate duration plays an important role in maximizing treatment efficacy in patients with MM, as it is directly correlated with patient survival [12]. Our previous real-world study using patients’ data from the Medical Data Vision (MDV) claims database in Japan found that the attrition rate was increased with each line of treatment (first line [transplant-conducted]-third line). Subsequently, the treatment duration became shorter in Japanese patients with MM from 2016 to 2020 (6.7-8.6 months) compared with 2003-2015 (8.8-10.0 months); the reduction was mostly seen in older adult patients (aged >80 y) [12]. Although the accurate reason for the shorter duration of therapy (DoT) was not established, it was hypothesized that older age, comorbid disease, availability of newer treatment regimens, safety and tolerability of treatments, and activities of daily living (ADLs) could have impacted the DoT in patients with MM [13-15]. Hence, accurate evaluation of the predictive factors that shorten the DoT could help in the treatment decision-making process.

Artificial intelligence (AI) using machine learning (ML) has been continuously used for the diagnosis and treatment of diseases. Using different tools and algorithms, AI eases the human experts’ tasks by computerization of the same. Informed evaluations are generated by detecting relationships in information [16]. Therefore, several studies have used different AI and ML algorithms for the diagnosis and risk stratification of MM using patient-level data as well as to create decision-making models [16-18].

This analysis is intended to identify predictive factors that shorten DoT at a threshold of 3, 6, and 12 months in patients with MM who had received first, second, and third-line treatments by applying an ML model using the patients’ data (type of first treatment and patient characteristics) from the MDV claims database.

## Methods

### Data Sources

This was a nationwide, retrospective observational cohort study conducted with secondary use of the Japan claims database over a 19-year period between 2003 and 2022. The study used longitudinal patient data from the MDV (MDV Co, Ltd) claims database, which is built over 450 Japanese hospital administrative data (covers approximately 26% of “Diagnosis Procedure Combination hospitals,” ie, the hospitals that perform higher than the lowest in the university hospital group), containing over 38 million patient data since the year 2003. The MDV database is a national, patient-level, anonymized, and longitudinal claims database including hospital claims, discharge summaries, hospitalization data, and outpatient and prescription data. The *ICD-10* (*International Statistical Classification of Diseases, Tenth Revision*) was used for disease diagnosis, and the medical procedures were coded using Japanese Procedure Codes. Hospitalized death and other clinical evaluations at discharge were available from the “discharge summary” data.

The study period was divided into a look-back period, treatment (time-at-risk) period, and follow-up period. The look-back period for covariates was the period from the first record of MM in the MDV database till the index date. Time-at-risk was the period from the start of the treatment to the end of the treatment for MM, while follow-up time was the period from the end of the treatment for MM to the last record registered in the MDV database. The index date was the record at the start of the treatment line in patients with transplant-ineligible NDMM or RRMM between 2003 and June 2021.

### Study Population

Male and female patients aged 18 years and older at index date with a confirmed diagnosis of MM (transplant-ineligible NDMM or RRMM), between 2003 and 2022, were included. Patients were required to have confirmed administration of any of the treatment regimens for first line for the transplant-ineligible NDMM or second line and third line for the RRMM. The unit of analysis (sample) in this study was an individual treatment of first line used for the patients who were transplant-ineligible NDMM or RRMM, second line, and third line. Patients whose DoT cannot be calculated were excluded from the ML analysis.

### Variables and Assessment

This study was conducted on 4848 samples for MM using 647 variables extracted from the MDV database. Variables were selected based on the collected opinions of local clinical experts and publications, and the results of a feasibility analysis. Final variables were determined after consulting local clinical experts on the results of the feasibility analysis and were broadly classified as patient, MM treatment-related, and MM treatment-related comorbidity management variables (Table 1). The primary end point was to identify the factors that correlate with the short DoT at the threshold of 3, 6, and 12 months. The DoT was defined as the time from the initiation of a line of treatment to the last day of the line of treatment.

**Table 1:** Feature variables extracted from the Medical Data Vision database to identify the important predictors associated with the duration of therapy.

Patient variables	MM^a^ treatment-related variables	MM treatment-related comorbidity management variables
Older age at the MM diagnosis (≥75 y, ≥80 y)^b^ Gender^b^ Height, weight^b^ Comorbidity (Charlson Index)^b^ eGFR^c^, blood pressure^b^ Leukocytes, neutrophils^b^ Lactate dehydrogenase^b^ Prothrombin time^b^ Independence (ADL^d^), Performance status^b^ Under nutrition^b^ Cardiac function^b^ Presence or absence of eating or swallowing dysfunction^b^ Frequency and history of depressive symptoms^b,e^ Frequency and history of use of antidepressants^b,e^ Frequency and history of use of hypnotics^b,e^ Year at diagnosis of MM^b^	Use of Bortezomib, Lenalidomide in previous treatment regimen^b^ Number of previous lines of regimens^b^ Use of triplet therapy in second line^e^ Use of oral drugs in the next line of regimens^e^ Stem cell transplantation conducted after the treatment regimen^e^ Steroid dose, timing of dose reduction^e^ Type of drug formulations in each regimen (eg, oral, with IV^f^, or with SC^g^)^e^ Type of hospital (ie, oncology-focused site), hospital size^b^ Type of treatment at molecule level (eg, IMiDs^h^-based, PI^i^-based, PI or IMiDs-based, anti-CD^j^28 mono, anti-CD38 combination, anti-SLAMF7^k^, anti-SLAMF7 combination, and other)^b^	Frequency and history of neutropenia management treatment (history of use of granulocyte-colony stimulating factor, frequency of administration, etc)^b,e^ Frequency and history of antibiotic use (levofloxacin)^b,e^ Frequency and history of prophylactic antibiotics^b,e^ History and frequency of antibiotic use for moderate to severe infections^b,e^ Frequency and history of pneumonia^b,e^ Frequency and history of thrombotic events^b,e^ Frequency and history of aspirin or direct oral anticoagulant use^e^

^a^MM: multiple myeloma.

^b^Variables used to develop machine learning models and understand the characteristics of clusters.

^c^eGFR: estimated glomerular filtration rate.

^d^ADL: activity of daily living.

^e^Variables used only to understand the characteristics of clusters (ie, variables not used to develop machine learning model).

^f^IV: intravenous.

^g^SC: subcutaneous.

^h^IMiDs: immunomodulatory drugs.

^i^PI: proteasome inhibitors.

^j^CD: cluster of differentiation.

^k^SLAM7: Signaling Lymphocytic Activation Molecule Family Member 7.

### Data Analysis

**Figure 1:**
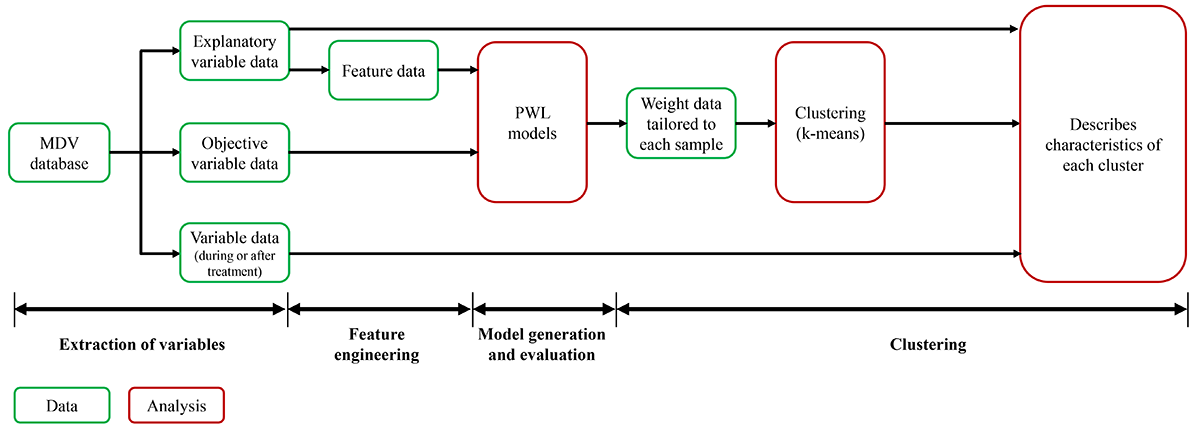
The data analysis pipeline used in the study. MDV: Medical Data Vision; PWL: point-wise linear.

Data analysis included 5 steps as discussed in Figure 1.

### Variable Extraction

ML models were constructed using 647 variables extracted from the MDV database to identify important predictors associated with DoT. The deep learning used in this study was applied to the exploratory research of biomarkers that are associated with the response of the immune checkpoint inhibitors [19,20]. The binary variables that represented DoT within 3, 6, or 12 months were categorized as target variables, while variables used to develop ML models and understand the characteristics of clusters listed in Table 1 were categorized as explanatory variables.

### Feature Engineering

All the feature variables were classified into continuous variables, binary variables, and nominal categorical variables. Continuous variables are normalized to have a mean of zero and an SD of 1 to eliminate bias due to differences in scale between variables. Binary variables were encoded to −1 or 1, and one-hot encoding (1 and −1) was used for nominal categorical variables [21]. The variables with missing values were imputed with 0 because the 0-value input would not change the output in the neural network models [22]. The target variables were set as a binary class and presented in 3 threshold patterns (0 for DoT <3 months, <6 months, or <12 months; and 1 for DoT ≥3 months, ≥6 months, or ≥12 months).

### Model Generation and Evaluation

**Figure 2:**
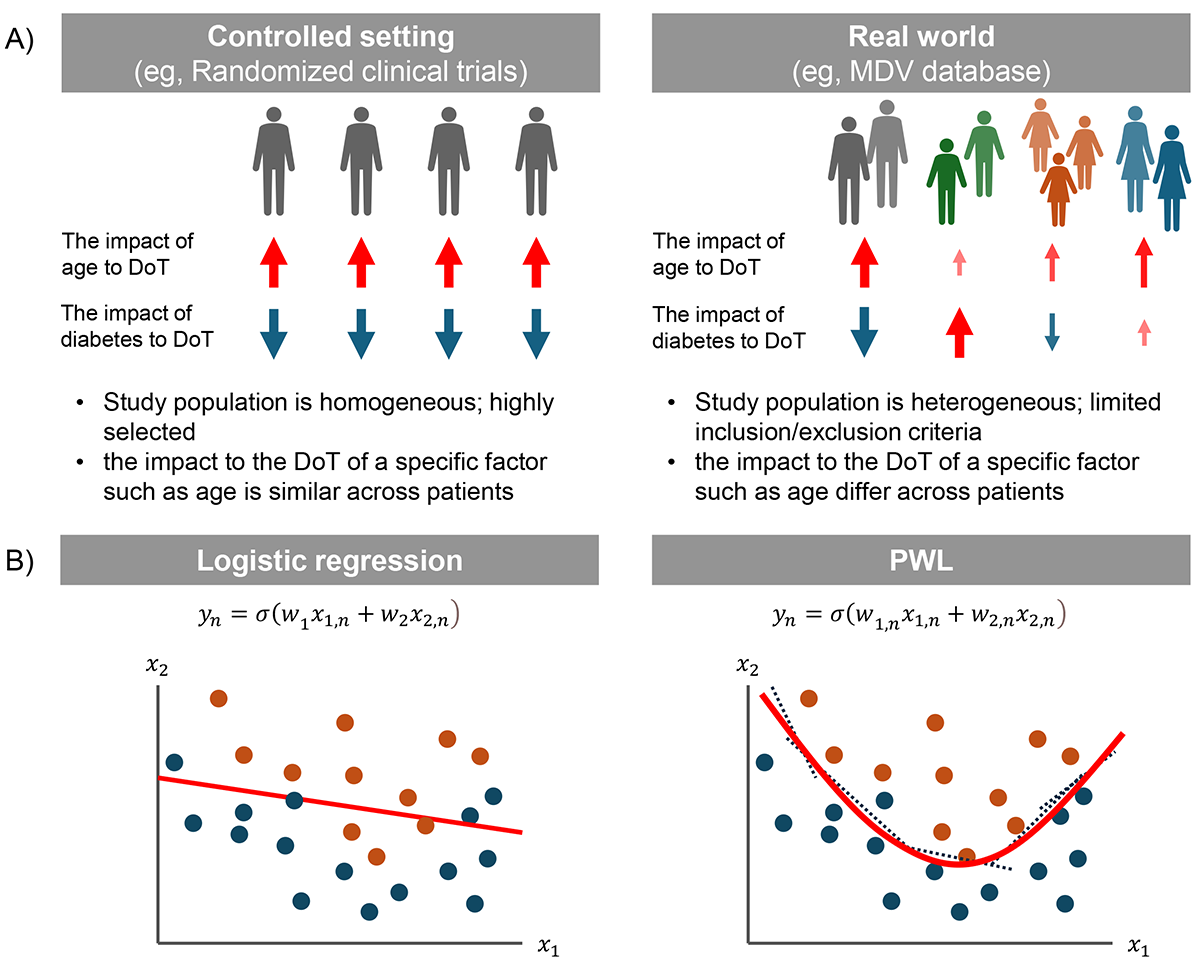
The overview of real-world data and machine learning algorithms. (A) The difference for controlled settings and real world. (B) The comparison of logistic regression model and PWL model. The orange and blue circles represent positive and negative class, respectively. The red lines represent boundaries defined by each model. The dashed lines represent sample-made logistic regression generated by PWL model. DoT: duration of treatment; PWL: point-wise linear model.

Unlike the randomized controlled trials used in clinical trials, the patients enrolled in the MDV database used in this study are a heterogeneous patient population (Figure 2A). In other words, the impacts of each variable on DoT differ across patients. We used point-wise linear (PWL) models to predict DoT. The PWL model was used as the explainable deep learning model, which generated a custom-made logistic regression (LR) model for each sample by a meta-learning approach (Figure 2B) [23]. Especially, although the LR model has a weight vector ***w*** for the original variable vector ***x*^(^*^n^*^)^** ([n] is the sample index), the PWL model has a point-wise weight vector ***w*^(^*^n^*^)^** for the original variable vector ***x*^(^*^n^*^)^** (Figure 2B). On the other hand, extreme gradient boosting (XGBoost) [24,25] is a decision-trees–based machine learning model capable of addressing patients’ heterogeneity. Although XGBoost achieves as high a performance as deep learning models do, in general, XGBoost cannot calculate the weight, that is, the impact on the DoT of each variable. In the finalization of the DoT prediction model, the area under the receiver operating characteristic curve (AUROC), recall, precision, and *F*_1_-score were calculated. The prediction performance of the PWL model was compared with the XGBoost (gradient-boosted decision trees) [24,25] and the LR model with a combination of L1 (Lasso) and L2 (Ridge) regularization terms (commonly known as elastic-net). The LR model is similar to PWL in that it explains feature importance by referencing the magnitude of weights (model parameters). The key difference between LR and PWL is that LR assigns a single weight coefficient to each feature across all samples, whereas PWL can assign different weights to the same feature for different samples. Consequently, PWL retains the capability to perform individualized factor analysis for each sample. The prediction performances of these models were calculated by the area under the curve (AUC) evaluated by 10-fold double cross-validation [26]. A “10-fold cross-validation” procedure was used, including 10 repeated attempts after classifying the data into 10 quadrants, with 1 quadrant for evaluation and the remaining 9 for learning. To avoid overlearning in hyperparameter tuning, the learning data were further divided into 10 pieces, 1 of which was used for tuning evaluation. The generalizability of the DoT prediction model was evaluated using the test set of the outer loop of 10-fold double cross-validation, and the datasets were reconstructed or hyperparameters were fine-tuned in case any significant difference (>0.1) was confirmed between the mean AUC in the test set and the training set.

### Clustering

A clustering analysis of 4848 individual samples was performed based on the similarity of the coefficients of custom-made LR models calculated from the PWL model to understand the number of patterns of the relationship between each sample and DoT (3, 6, and 12 months). All the samples were classified into subgroups using the regression coefficient vector tailored to each sample in the PWL model. Cluster analysis was conducted using the k-means method [27,28], and the number of clusters (k) was determined by the Elbow method [29]. The characteristics of each cluster were analyzed by using all the variables listed in Table 1. The *P* values for continuous variables were assessed by *t* test and 1-way ANOVA, while the *P* values for categorical variables were assessed by chi-square test. To understand the characteristics within a particular cluster, we categorized all 4848 samples into 2 groups—those predicted not to continue treatment after the respective DoT threshold and those predicted to continue treatment after the respective DoT threshold. Continuous variables and categorical variables significantly different between the groups were assessed by 1-way ANOVA and chi-square test, respectively. The *P* values were adjusted by the Bonferroni correction method.

### Ethical Considerations

All data used in this study were deidentified and anonymized patient records obtained from the MDV database for secondary research purposes. In accordance with the Ethical Guidelines for Epidemiological Research issued by the Japanese Ministry of Health, Labor, and Welfare, the study did not require informed consent or approval from an Institutional Review Board [30].

## Results

### Baseline Demographics and Clinical Characteristics

This analysis was conducted in 2762 patients (4848 individual samples) with MM. Of these, 1450 (52.5%) were male; 2079 (75.3%) patients were included during the index year 2016-2021. At the first MM diagnosis, the mean age, Charlson Comorbidity Index (CCI) score, and ADL score were 69.6 (SD 11.8) years, 0.5 (SD 1.1), and 14.6 (SD 7.2), respectively (Table 2).

**Table 2:** Baseline demographics and clinical characteristics of patients with multiple myeloma.

Characteristics			Overall (N=2762)
Age at first MM^a^ diagnosis, mean (SD)			69.6 (11.8)
**Age groups at first MM diagnosis (y), n (%)**			
	18-59	540 (19.6)	
	60-64	344 (12.5)	
	≥65	1878 (68)	
**Sex, n (%)**			
	Male	1450 (52.5)	
	Female	1312 (47.5)	
**Index year, n (%)**			
	2003-2015	683 (24.7)	
	2016-2021	2079 (75.3)	
	CCI^b^ score at first MM diagnosis, mean (SD)	0.5 (1.1)	
**CCI score at first MM diagnosis, n (%)**			
	0	2121 (76.8)	
	1	281 (10.2)	
	2	177 (6.4)	
	3	99 (3.6)	
	≥4	84 (3)	
ADL^c^ score at first MM diagnosis, mean (SD)^d,e^			14.6 (7.2)
**ADL score at first MM diagnosis, n (%)^d,e^**			
	0	111 (8.2)	
	1	38 (2.8)	
	2	31 (2.3)	
	3	18 (1.3)	
	4-19	466 (33.3)	
	20	694 (51.1)	
Degree of care requirement at diagnosis of MM, mean (SD)^d,f^			0.4 (1.2)
**Degree of care requirement at diagnosis of MM, n (%)^d,f^**			
	0	564 (86.5)	
	1	24 (3.7)	
	2	14 (2.1)	
	3	16 (2.5)	
	≥4	34 (5.2)	
**Tube and parenteral nutrition at first MM diagnosis, n (%)^d,g^**			
	0	214 (92.2)	
	1	17 (7.3)	
	2	1 (0.4)	
**Stage of MM at first MM diagnosis^d,h^**			
	Mean (SD)	5.0 (0.0)	
	5, n (%)	3 (100)	
NYHA at first MM diagnosis, mean (SD)^d,i^			2.1 (1.1)
**NYHA at first MM diagnosis, n (%)^d,i^**			
	0	2 (4.8)	
	1	15 (35.7)	
	2	7 (16.7)	
	3	14 (33.3)	
	4	4 (9.5)	
**Cancer at first MM diagnosis, n (%)^d,j^**			
	Primary	706 (97.8)	
	Recurrence	16 (2.2)	

^a^MM: multiple myeloma.

^b^CCI: Charlson Comorbidity Index.

^c^ADL: activity of daily living.

^d^Missing data were present and these entries were not included in the table.

^e^The number of patients with missing values was 1404.

^f^The number of patients with missing values was 2110.

^g^The number of patients with missing values was 2530.

^h^The number of patients with missing values was 2759.

^i^The number of patients with missing values was 2720.

^j^The number of patients with missing values was 2040.

### Prediction Performance

**Figure 3:**
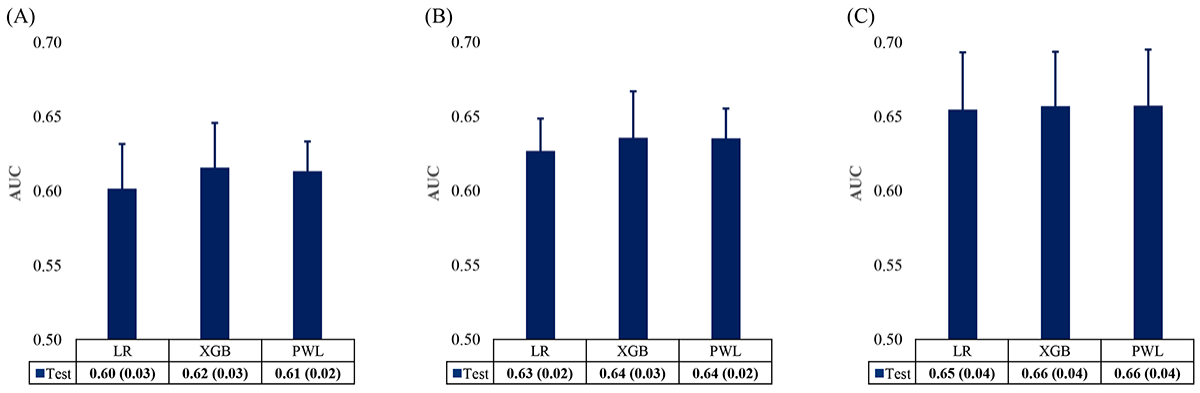
Predictive performance of different models to predict duration of therapy (DoT) thresholds. (A) DoT at 3 months, (B) DoT at 6 months, and (C) DoT at 12 months. The graphs represent mean (SD) of area under the curve in 10-fold double cross-validation. LR: regularized logistic regression; PWL: point-wise linear model; XGB: extreme gradient boosting.

The AUROC score of the PWL model to predict DoT at 3, 6, and 12 months was 0.61, 0.64, and 0.66, respectively. The corresponding values for the XGBoost model were 0.62, 0.64, and 0.66, respectively. The PWL model with the target variable DoT at 12 months performed the best; however, no model reached an AUC>0.7 (Figure 3). Moreover, the slightly smaller SD in PWL suggested more consistent performance across different folds of cross-validation, making it the model of choice for this analysis. The recall, precision, and *F*_1_-score of all models are presented in the supplementary material (Multimedia Appendix 1). The precision and *F*_1_-score were decreased when the DoT length increased, and the thresholds shortened.

### Cluster Analysis

#### Overview

The PWL model generated regression models for each patient, allowing the clustering of patient populations based on the similarity of the coefficients of the regression models. When classified into different clusters, it was found that there were multiple populations with diverse groups of factors affecting the prediction. The samples were categorized into 2 clusters (cluster A and cluster B) at DoT of 3 months and 3 clusters (cluster A, cluster B, and cluster C) at each DoT of 6 months and DoT of 12 months. The baseline demographics and clinical characteristics of 4848 individual samples categorized as per different clusters at DoT of 3, 6, and 12 months are presented in Multimedia Appendix 2. In addition, we presented the distribution of each cluster in the 2 major components of the variables transformed by principal component analysis in the supplementary materials (Multimedia Appendix 3).

#### At DoT of 3 Months

**Figure 4:**
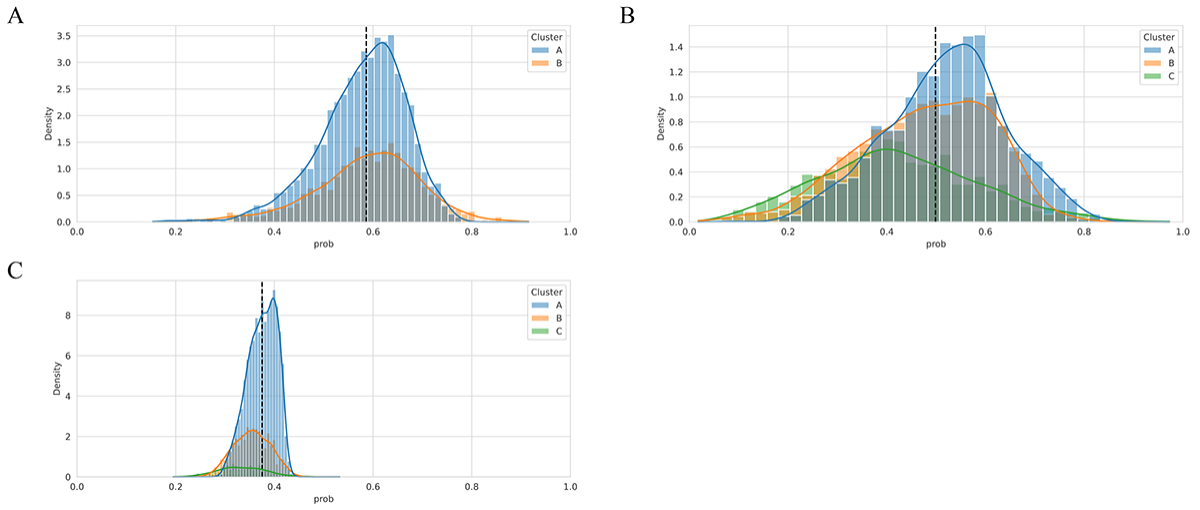
Detailed cluster analysis. (A) Duration of treatment (DoT) at 3 months, (B) DoT at 6 months, (C) DoT at 12 months. The dashed lines indicate respective classification thresholds (3, 6, and 12 months, respectively, for A, B, and C). The group to the left of the dotted line indicates those predicted not to continue treatment after respective DoT threshold, and the group to the right of the dotted line indicates those predicted to continue treatment after respective DoT thresholds (3, 6, and 12 months, respectively, for A, B, and C).

Of the 4848 samples, 3287 (67.8%) samples were included in cluster A and 1561 (32.2%) were included in cluster B. Compared with cluster A, cluster B had more patients with age <75 years, male sex, index year 2003-2015, and CCI ≥4 (Multimedia Appendix 2). In the probability analysis, cluster B reported a lower median probability than cluster A, with pointed and extended left tails **(**Figure 4A). From Figure 4A, it can be estimated that many patients in cluster B might be in poor condition, but some were predicted to continue treatment for ≥3 months. Compared with cluster A, patients in cluster B had significantly more lines of previous therapy (0.5 vs 1.6; *P*<.001), higher pretreatment CCI (2.2 vs 2.5; *P*<.001), and a higher level of care requirement at the time of diagnosis of MM (0.4 vs 0.7; *P=*.001). Additionally, cluster B had significantly (*P*<.001) higher proportions of patients with pneumonia, depression, cancer, gastroesophageal reflux disease (GERD), other bowel dysfunctions, primary hypertension, pain, purine and pyrimidine metabolism disorders, and type 2 diabetes mellitus (T2DM) than cluster A (Multimedia Appendix 2).

#### At DoT of 6 Months

Of the 4848 samples, 2011 (41.5%) were included in cluster A, 1736 (35.8%) were included in cluster B, and 1101 (22.7%) were included in cluster C. Compared with cluster A and B, cluster C had more patients with age <75 years, female sex, the index year 2016-2021, and CCI ≥4 (Multimedia Appendix 2). In the probability analysis, cluster C reported a lower median probability than clusters A and B, with flat and extended right tails (Figure 4B). From Figure 4B, it can be estimated that many patients in cluster C had many comorbidities, but some were predicted to continue treatment for ≥6 months. Patients in cluster B had significantly lower estimated glomerular filtration rate (*P*<.001) and higher neutrophils (*P*<.001), while cluster C had significantly higher pretreatment CCI (*P*<.001) compared to other clusters. Additionally, cluster C had significantly (*P*<.001) higher proportions of patients with pneumonia, depression, cancer, GERD, other bowel dysfunction, primary hypertension, pain, purine and pyrimidine metabolism disorders, and T2DM than clusters A and B (Multimedia Appendix 2).

#### At DoT of 12 Months

Of the 4848 samples, 3425 (70.6%) were included in cluster A, 1123 (23.2%) were included in cluster B, and 300 (6.2%) were included in cluster C. Compared with cluster A and B, cluster C had more patients with male sex, index year 2016-2021, and CCI ≥4 (Multimedia Appendix 2). In the probability analysis, cluster C reported a lower median probability than cluster A and B, with peaked and extended right tails (Figure 4C). From Figure 4C, it can be estimated that many patients in cluster C were in poor condition, but some were predicted to continue treatment for ≥12 months. Cluster C had significantly higher neutrophils (*P*<.001), leukocytes (*P*<.001), lactate dehydrogenase (*P*<.001), previous treatment lines (*P*<.001), pretreatment CCI (*P*<.001), degree of care requirement at diagnosis of MM (*P*<.001), and pretreatment tube and parenteral nutrition (*P*<.001) among clusters (Multimedia Appendix 2). Additionally, cluster C had a statistically (*P*<.001) higher proportion of patients with comorbidities, including depression, pneumonia, cancer, GERD, other bowel dysfunction, primary hypertension, pain, purine and pyrimidine metabolism disorders, and T2DM, than cluster A and B.

### Characteristics of Each Cluster Before and After the DoT Threshold

All 4848 samples were categorized before and after each DoT threshold (3, 6, and 12 months) to determine the factors responsible for shortened DoT. As cluster B at DoT of 3 months and cluster C at DoT of 6 and 12 months were predicted to continue the treatment, patients from these clusters were further divided into those predicted to continue treatment after the DoT threshold, and those predicted to discontinue treatment within the DoT threshold, and the incidence of events during and after treatment was compared. There were a lesser number of treatments included in DoT <3 months than DoT ≥3 months (n=1859, 38.3% vs n=2989, 61.7%), while DoT <6 (n=2753, 56.8%) and <12 months (n=3672, 75.7%) had a higher number of treatments compared with DoT ≥6 (n=2095, 43.2%) and ≥12 months (n=1176, 24.3%), respectively. In cluster B, a significantly (*P*<.01) higher percentage of patients used immunomodulatory drugs (IMiDs) and used or maintained aspirin during MM therapy in DoT ≥3 than in DoT <3 months group. However, the rate of subsequent transplantation in the next treatment was significantly (*P*<.01) higher in DoT <3 than DoT ≥3 months group. Similarly, in cluster C, a significantly (*P*<.01) higher percentage of patients used IMiDs or aspirin during MM therapy in the DoT ≥6 months than in the DoT <6 months group. In cluster C, a significantly (*P*<.01) higher percentage of patients used IMiDs during MM therapy in DoT ≥12 months than in the DoT <12 months group (Table 3). A summary of the key clinical traits for each cluster after the DoT threshold analysis is presented in Table 4.

**Table 3:** The characteristics between before and after treatment thresholds, as per the duration of therapy.

Medications	Cluster B				Cluster C				Cluster C		
	<3 months, n/N (%)	≥3 months, n/N (%)	Adjusted *P* value	<6 months, n/N (%)		≥6 months, n/N (%)	Adjusted *P* value	<12 months, n/N (%)		≥12 months, n/N (%)	Adjusted *P* value
Samples	1859/4848 (38.3)	2989/4848 (61.7)	—^a^	2753/4848 (56.8)		2095/4848 (43.2)	—	3672/4848 (75.7)		1176/4848 (24.3)	—
Use of IMiDs^b^	261/714 (36.6)	624/847 (73.7)	<.01	311/763 (40.8)		238/338 (70.4)	<.01	91/240 (37.9)		43/60 (71.7)	<.01
Use of aspirin	206/714 (28.9)	431/847 (50.9)	<.01	244/763 (32.0)		170/338 (50.3)	<.01	69/240 (28.8)		32/60 (53.3)	.77
Continue aspirin	125/714 (17.5)	236/847 (27.9)	<.01	—		—	—	—		—	—
Subsequent transplant	90/714 (12.6)	19/847 (2.2)	<.01	—		—	—	—		—	—

^a^Not applicable.

^b^IMiD: immunomodulatory drug.

**Table 4:** A summary of the key clinical characteristics distinguishing clusters.

Cluster	DoT^a^ threshold	Key characteristics	CCI^b^≥4, n/N (%)	IMiD^c^ use (≥DoT), n/N (%)	Aspirin use (≥DoT), n/N (%)
Cluster B	3 months	Higher comorbidity, more prior therapy	393/1561 (25.2)	624/847 (73.7)	431/847 (50.9)
Cluster C	6 months	High comorbidity, more comorbid events	383/1101 (34.8)	238/338 (70.4)	170/338 (50.3)
Cluster C	12 months	High neutrophils, LD^d^, leukocytes	127/300 (42.3)	43/60 (71.7)	32/60 (53.3)

^a^DoT: duration of therapy.

^b^CCI: Charlson Comorbidity Index.

^c^IMiD: immunomodulatory drug.

^d^LD: lactate dehydrogenase.

## Discussion

### Principal Findings

This retrospective real-world observational study using longitudinal patients’ data from the nationwide MDV claims database analyzed the predictive factors that shorten DoT in Japanese patients with MM over a 19-year period (2003-2022). Additionally, this analysis used ML models to determine the factors responsible for shorter DoT using information, such as type of treatment and patient characteristics from the MDV claims database, categorizing all the data in different clusters at 3, 6, and 12 months. Finally, a clustering analysis was conducted to investigate the characteristics of the sample in each cluster and the relationship between predicted DoT and the comorbidity management before and after the treatment thresholds (3, 6, and 12 months).

Patients in cluster B than cluster A (at 3 months), and patients in cluster C than cluster A and B (at 6 and 12 months) were supposed to be in poor condition as the number of treatment lines and pretreatment CCI were high; patients had more comorbidities, and elevated laboratory parameters. Cluster B (3-month DoT) and cluster C (6- and 12-month DoT) consistently captured subgroups with high comorbidity burden and elevated pretreatment CCI, yet demonstrated longer treatment durations. Additionally, the use of IMiDs for MM treatment was significantly higher in patients who met predicted DoT at each threshold versus those who did not. In contrast to the clinical findings, the data from this study indicate that the choice of drug, irrespective of whether it is used for induction or maintenance therapy, affects the expected DoT.

### Comparison With Previous Work

From our recent real-world study in Japan, it was found that the DoT decreased continuously with increasing the line of treatment in patients with MM, especially after 2016 when several newer therapy options were in force [12]. In clinical practice, treatment may be discontinued for various reasons; however, for the desired prognosis, it is important to continue the medications for an appropriate duration. The DoT has a strong association with the clinical outcomes and survival in patients with MM [31,32]; therefore, it was inevitable to determine the factors affecting the shortened DoT. Although it was evident that the availability of new drugs might increase the treatment options, making it easier to change treatment regimens, the huge pattern of treatment makes it difficult to identify the cause of shortened DoT. Therefore, this analysis was conducted using the national MDV claims database to determine the predictive factors that could help clinicians in the decision-making process while considering the management of MM. Additionally, this study found the characteristics of patients (age, CCI, comorbidity, treatment lines, laboratory parameters, and care requirement) who had shorter DoT as well as the characteristics of patients with frequent treatment change, which reflects the actual picture of clinical practice. This information is critical to continually improving MM management, achieving optimal patient care, and understanding the burden of disease and health services available to Japanese patients with MM in the real world.

In line with the published evidence [33,34], our study compared the prediction performance of the PWL model with the XGBoost and the LR models. The AUROC values of constructed models were all between 0.6 and 0.7 (Figure 2). Values between 0.6 and 0.7 indicate satisfactory performance [35-37]. The ranges observed in this study are consistent with those reported in previous real-world data–based modeling studies [33,38,39]. Administrative claims data often face inherent data limitations, such as the absence of genomic or staging variables, which can constrain predictive performance. In real-world settings, such models can still provide clinically relevant insights for subgroup identification and hypothesis generation. Nonetheless, models built on such data can still yield clinically relevant insights—particularly for subgroup identification and hypothesis generation in real-world settings. In our study, we generated the models using the variables required in the claims database. For improvements in prediction performance, the use of MM-specific laboratory parameters might be required. Furthermore, in our study, the PWL model showed greater AUROC than the linear regression model at the DoT thresholds of 3, 6, and 12 months. However, these scores were the differences within the error bars between PWL and XGBoost models at each DoT threshold.

### DoT Assessment Using PWL Models

DoT predictive models in our study were specifically designed to forecast the DoT in patients with MM, rather than to evaluate clinical effectiveness between specific treatment groups. Due to this focus, these models were not intended for direct application in clinical decision-making or disease diagnosis. Consequently, we did not perform propensity score matching or sensitivity analysis, which are typically used in studies evaluating treatment efficacy. Instead, our efforts were concentrated on carefully developing and comparing the PWL model with the XGBoost and LR models to thoroughly assess the factors influencing DoT. The PWL model differs from LR by generating a separate set of regression coefficients for each individual sample, rather than using a single global set. This approach allows identification of factors that are most relevant for each patient, which may enhance interpretability and personalization in real-world datasets where treatment decisions and outcomes are highly individualized. Unlike randomized controlled trials, the MDV claim database includes a diverse and unselected patient population, without adjustments for specific demographics or conditions. This makes the data more representative of real-world variability. Multiple patterns can thus be anticipated based on the combination of variables that affect DoT. The LR model cannot recognize multiple patterns because the model has only a single regression coefficient to a variable for the whole sample. On the other hand, since the PWL model has a custom-made regression coefficient, it is possible to perform individual analysis for each sample. Therefore, we performed the cluster analysis using the regression coefficient vector tailored to each sample in the PWL model to understand the number of patterns. The PWL model enabled case-level insights, highlighting which patient features most influenced predicted treatment duration. Such interpretability is especially valuable in clinical settings, where understanding the rationale behind risk stratification supports shared decision-making. In our study, the k-means method was used for cluster analysis to classify all the samples into subgroups, and the Elbow method was used to determine the number of clusters. The k-means method is an unsupervised ML approach widely used in research for clustering samples based on similarities [28,40]. Additionally, the k-means method could substantiate heterogeneous longitudinal data into distinct, homogeneous clusters [27,28]. The Elbow method is an established method to determine the appropriate number of clusters that rides the clustering algorithm many times with different values of k (number of clusters) [41], and defines the optimal number of clusters to efficiently categorize the data [42,43].

From the baseline demographics and predictors of cluster assignment, it was found that patients in cluster B at DoT of 3 months and cluster C at DoT of 6 and 12 months, compared with other clusters, had advanced disease and continued the treatment beyond the threshold duration. Interestingly, we observed that in these clusters, more patients had CCI ≥4, ≥1 line of treatment, and comorbidities (pneumonia, depression, cancer, GERD, and other bowel dysfunction) than in other clusters at each DoT threshold. Additionally, the laboratory parameters such as estimated glomerular filtration rate (MDV specific), leukocytes, neutrophils, and lactate dehydrogenase were significantly higher in the cluster C than the cluster A and B at DoT 12 months. An all-inclusive assessment of comorbidities and overall status (with the help of laboratory parameters) may help to determine a patient’s suitability for appropriate therapy as well as the adverse effects that may occur. Altogether, the results from this study support the understanding that the patient’s condition and management-related factors may be associated with shortened DoT, consistent with clinical findings [12,44,45]. Evidence suggests that severe disease, older age, and comorbidities are the prognostic factors for poor survival in patients with MM [46,47]. Although it was not in the scope of this analysis to comment on the causal relationship between predictive factors and patient survival, and their effect on longitudinal outcomes and DoT, our findings validate a depiction of shortened DoT in those with reduced disease burden. This is consistent with the findings of the retrospective study conducted by Ailawadhi S et al [32], which reported that each additional month of therapy was associated with a reduced probability of disease progression and death at 2 years. Similar results were also observed in a pooled analysis of 3 phase III trials that evaluated the advantage of novel agent-based continuous therapy versus fixed DoT in patients with newly diagnosed myeloma [48].

To understand the characteristics of the clusters, we analyzed the relationship between the explanatory variables and the flag that indicated the samples belonged to each cluster. Additionally, we calculated the association between the variables during and the flag to investigate the tendency to the event during and after treatment. The significance was detected by the test for noncorrelation at a significance level false discovery rate of <0.05 using the Benjamini-Hochberg method. The Benjamini-Hochberg method is a validated tool used in multiple correlation analyses that could decrease the false discovery rate [19].

Significantly, more patients used IMiDs and aspirin beyond the treatment thresholds (both ≥3 [cluster B] and ≥6 months [cluster C]) compared with before treatment thresholds (<3 and <6 months), and in cluster C at DoT ≥12 months, a higher proportion of patients used IMiDs during MM therapy compared with DoT <12 months group. The use of IMiDs was significantly higher when comparing sample populations predicted as DoT of <3, <6, and <12 months with those who did not. However, this observational association may reflect confounding factors, such as patient eligibility, tolerability, or physician preference, and does not imply causation. In patients with MM, lenalidomide, one of the commonly used IMiDs, is sometimes used in maintenance therapy, and the results from this study suggest that IMiD therapies are associated with treatment continuation for MM in patients with high CCI, severe disease, and comorbidities. Furthermore, these results imply that the use of appropriate IMiD may impact DoT in patients with MM. Since the treatment choices for patients with MM depend on several factors, including the type of previous therapy and comorbidity, these data may assist the clinical decision-making on whether IMiDs should be used in these patients. Similar to IMiD use, the management of thromboprophylaxis was also associated with treatment continuation for MM in patients with high CCI.

### Factors Identified for Shortened DoT

Our analysis identified several key factors that might be associated with shortened DoT for patients with MM. The first factor is the patient’s disease status—patients with poorer health status (CCI ≥4, ≥1 previous line of treatment, and comorbidities including pneumonia, depression, cancer, GERD, or bowel dysfunction), along with elevated laboratory parameters, showed longer DoT. Second was the management-related factors, including use of IMiDs and management of thromboprophylaxis: patients who were treated with IMiDs for MM showed extended DoT.

### Strengths and Limitations

The strengths of the study include (1) this study is a precedent of data-driven research to understand the characteristics of patients with MM whose DoT was shortened; (2) by using AI, based on ML techniques, we were able to build a prediction model using many factors that are difficult to build with a conventional statistical model; (3) in this study, subgrouping was automatically done by the ML-based DoT predictive models as subgrouping analysis is required when analyzing real-world data due to heterogeneity of the patient population; (4) this study used the latest data from nationwide MDV claims database which was based on Japanese general population, and represented real-world clinical practice.

This study also has certain limitations. First, the database is restricted to the Japanese population; hence, caution should be taken while generalizing the results. This database only included the data of the insured population, and it was impossible to follow patients outside of the hospital or those who switched hospitals. Not all laboratory tests were conducted in the entire patient population, which again restricts the generalizability of the findings. Furthermore, since the cause of death was unavailable, it could not be determined whether the shorter DoT was due to treatment failure, toxicity, patient preference, or unrelated mortality. Additionally, death was captured only for hospitalized patients, and important prognostic factors for MM, such as cytogenetic profile, R-International Staging System stage, and presence of all plasmacytomas, were not collected. This may affect the clinical granularity and predictive robustness of the models. Their inclusion may further enhance both robustness and interpretability of future predictive tools. We partially addressed this by incorporating surrogate measures, such as CCI and ADL scores. Furthermore, it is difficult to evaluate the causal relationship between predictive factors and DoT, even if the relationship between the factors and shortening of DoT is assumed, and the presence of unknown confounding that affects the continuation of treatment cannot be ruled out.

### Conclusion

This study is the first to apply ML to assess predictive factors for shortened DoT in Japanese patients with MM. Using the PWL model, an explainable deep learning method, we identified key patient and management factors influencing DoT. These insights can contribute to improving patient management in hospitals, optimizing care, and enhancing understanding of the burden of disease and health services for Japanese patients with MM in real-world settings. Further statistical analysis is needed to refine patient segmentation and ensure the effective application of these insights in clinical environments.

## Data Availability

The data that support the findings of this study were derived from the MDV database. Restrictions apply to the availability of these data, which were used under license for this study.

## Appendix

Multimedia Appendix 1 Table S1: The precision, recall and F1 score of all models.

Multimedia Appendix 2 Table S2: Details of characteristics of patients with MM treatment stratified by clusters at DoT 3, 6 and 12 months. Missing values from total samples: a4428, b4217, c4221, d4420, e4254, f3624. CCI, Charlson Comorbidity Index; DoC, degree of care; DoT, duration of treatment; eGFR, estimated glomerular filtration rate; GERD, gastro esophageal reflux disease; HTN, hypertension; LoT, line of treatment; MDV, Medical Data Vision; MM, multiple myeloma; T2DM, type 2 diabetes mellitus.

Multimedia Appendix 3 Two-dimensional cluster analysis using PCA at DoT of 3, 6 and 12 months Dimensional compression was performed by PCA to confirm the relationship between clusters in two-dimensional plots. Each result was drawn to correspond to a previously defined cluster. DoT, duration of treatment; PCA, principal component analysis.

## Abbreviations

ADL: activity of daily living
AI: artificial intelligence
AUC: area under the curve
AUROC: area under the receiver operating characteristic curve
CCI: Charlson Comorbidity Index
DoT: duration of therapy
ICD-10: International Statistical Classification of Diseases, Tenth Revision
GERD: gastroesophageal reflux disease
IMiD: immunomodulatory drugs
LR: logistic regression
MDV: Medical Data Vision
ML: machine learning
MM: multiple myeloma
NDMM: newly diagnosed multiple myeloma
PWL: point-wise linear
RRMM: relapsed or refractory multiple myeloma
T2DM: type 2 diabetes mellitus
XGBoost: extreme gradient boosting
