# Supplementary material for: Assessment of Predictive Factors That Shorten Duration of Treatment in Patients With Multiple Myeloma Using AI: Real-World Longitudinal Study Using Data From Medical Data Vision Claims Database
Source: JMIR Cancer. 2026 Feb 19;12:e75586. doi: 10.2196/75586 (PMC12963979; doi:10.2196/75586)
Supplement: Multimedia Appendix 1 [file cancer_v12i1e75586_app1.docx]

**Table S1: The precision, recall and F1 score of all models.**

| **Parameters** |  | **Logistic regression model** | **XGBoost model** | **PWL model** |
| --- | --- | --- | --- | --- |
| Precision | DoT at 3 months | 0.69 ± 0.02 | 0.70 ± 0.03 | 0.70 ± 0.02 |
|  | DoT at 6 months | 0.53 ± 0.03 | 0.54 ± 0.03 | 0.54 ± 0.02 |
|  | DoT at 12 months | 0.35 ± 0.04 | 0.35 ± 0.03 | 0.35 ± 0.04 |
| Recall | DoT at 3 months | 0.60 ± 0.05 | 0.58 ± 0.06 | 0.61 ± 0.03 |
|  | DoT at 6 months | 0.62 ± 0.05 | 0.65 ± 0.05 | 0.62 ± 0.04 |
|  | DoT at 12 months | 0.64 ± 0.05 | 0.63 ± 0.07 | 0.64 ± 0.04 |
| F1 score | DoT at 3 months | 0.64 ± 0.03 | 0.63 ± 0.04 | 0.65 ± 0.02 |
|  | DoT at 6 months | 0.57 ± 0.03 | 0.59 ± 0.02 | 0.58 ± 0.02 |
|  | DoT at 12 months | 0.45 ± 0.04 | 0.45 ± 0.03 | 0.45 ± 0.04 |

The data represents the average precision or recall or F1 score ± standard deviation in 10-fold DCV.

DCV, double cross validation; DoT, duration of treatment.
