# Supplementary material for: Assessment of Predictive Factors That Shorten Duration of Treatment in Patients With Multiple Myeloma Using AI: Real-World Longitudinal Study Using Data From Medical Data Vision Claims Database
Source: JMIR Cancer. 2026 Feb 19;12:e75586. doi: 10.2196/75586 (PMC12963979; doi:10.2196/75586)
Supplement: Multimedia Appendix 2 [file cancer_v12i1e75586_app2.docx]

**Appendix**

**Table S1: The precision, recall and F1 score of all models.**

| **Parameters** |  | **Logistic regression model** | **XGBoost model** | **PWL model** |
| --- | --- | --- | --- | --- |
| Precision | DoT at 3 months | 0.69 ± 0.02 | 0.70 ± 0.03 | 0.70 ± 0.02 |
|  | DoT at 6 months | 0.53 ± 0.03 | 0.54 ± 0.03 | 0.54 ± 0.02 |
|  | DoT at 12 months | 0.35 ± 0.04 | 0.35 ± 0.03 | 0.35 ± 0.04 |
| Recall | DoT at 3 months | 0.60 ± 0.05 | 0.58 ± 0.06 | 0.61 ± 0.03 |
|  | DoT at 6 months | 0.62 ± 0.05 | 0.65 ± 0.05 | 0.62 ± 0.04 |
|  | DoT at 12 months | 0.64 ± 0.05 | 0.63 ± 0.07 | 0.64 ± 0.04 |
| F1 score | DoT at 3 months | 0.64 ± 0.03 | 0.63 ± 0.04 | 0.65 ± 0.02 |
|  | DoT at 6 months | 0.57 ± 0.03 | 0.59 ± 0.02 | 0.58 ± 0.02 |
|  | DoT at 12 months | 0.45 ± 0.04 | 0.45 ± 0.03 | 0.45 ± 0.04 |

The data represents the average precision or recall or F1 score ± standard deviation in 10-fold DCV.

DCV, double cross validation; DoT, duration of treatment.

**Table S2:** **Details of characteristics of patients with MM treatment stratified by clusters at DoT 3, 6 and 12 months.**

| **Characteristics** | **Total** | **At DoT 3 months** | | | **At DoT 6 months** | | | | **At DoT 12 months** | | | |
| --- | --- | --- | --- | --- | --- | --- | --- | --- | --- | --- | --- | --- |
| **Clusters** |  | **A** | **B** | ***P*-value** | **A** | **B** | **C** | ***P*-value** | **A** | **B** | **C** | ***P*-value** |
|  |  |  |  |  |  |  |  |  |  |  |  |  |
| **Samples (n)** | 4848 | 3287 | 1561 | - | 2011 | 1736 | 1101 | - | 3425 | 1123 | 300 | - |
| **Age, n (%)** | | | | | | | | | | | | |
| <75 years | 2980 (61.5) | 1830 (55.7) | 1150 (73.7) | <.001 | 1253 (62.3) | 954 (55.0) | 773 (70.2) | <.001 | 2085 (60.9) | 712 (63.4) | 183 (61.0) | .32 |
| ≥75 years | 1868 (38.5) | 1457 (44.3) | 411 (26.3) |  | 758 (37.7) | 782 (45.0) | 328 (29.8) |  | 1340 (39.1) | 411 (36.6) | 117 (39.0) |  |
| **Gender, n (%)** | | | | | | | | | | | | |
| Male | 2568 (53.0) | 1690 (51.4) | 878 (56.2) | .002 | 1110 (55.2) | 957 (55.1) | 501 (45.5) | <.001 | 1810 (52.8) | 596 (53.1) | 162 (54.0) | .93 |
| Female | 2280 (47.0) | 1597 (48.6) | 683 (43.8) |  | 901 (44.8) | 779 (44.9) | 600 (54.5) |  | 1615 (47.2) | 527 (46.9) | 138 (46.0) |  |
| **First MM diagnosis at index year, n (%)** | | | | | | | | | | | | |
| 2003-2015 | 1269 (26.2) | 800 (24.3) | 469 (30.0) | <.001 | 508 (25.3) | 560 (32.3) | 201 (18.3) | <.001 | 945 (27.6) | 268 (23.9) | 56 (18.7) | .05 |
| 2016-2021 | 3579 (73.8) | 2487 (75.7) | 1092 (70.0) |  | 1503 (74.7) | 1176 (67.7) | 900 (81.7) |  | 2480 (72.4) | 855 (76.1) | 244 (81.3) |  |
| **CCI, mean±SD** | 2.3±2.5 | 2.2±2.4 | 2.5±2.7 | <.001 | 1.6±1.9 | 2.5±2.5 | 3.2±3.0 | <.001 | 2.1±2.3 | 2.6±2.8 | 3.6±3.0 | <.001 |
| **CCI, n (%)** | | | | | | | | | | | | |
| 0 | 1286 (26.5) | 888 (27.0) | 398 (25.5) | <.001 | 673 (33.5) | 410 (23.6) | 203 (18.4) | <.001 | 976 (28.5) | 271 (24.1) | 39 (13.0) | <.001 |
| 1 | 1035 (21.3) | 715 (21.8) | 320 (20.5) |  | 506 (25.2) | 340 (19.6) | 189 (17.2) |  | 762 (22.2) | 227 (20.2) | 46 (15.3) |  |
| 2 | 844 (17.4) | 574 (17.5) | 270 (17.3) |  | 365 (18.2) | 298 (17.2) | 181 (16.4) |  | 597 (17.4) | 185 (16.5) | 62 (20.7) |  |
| 3 | 599 (12.4) | 419 (12.7) | 180 (11.5) |  | 216 (10.7) | 238 (13.7) | 145 (13.2) |  | 421 (12.3) | 152 (13.5) | 26 (8.7) |  |
| ≥4 | 1084 (22.4) | 691 (21.0) | 393 (25.2) |  | 251 (12.4) | 450 (25.9) | 383 (34.8) |  | 669 (19.6) | 288 (25.7) | 127 (42.3) |  |
| **Number of previous LoT, mean±SD** | 0.9±0.8 | 0.5±0.6 | 1.6±0.5 | <.001 | 0.5±0.7 | 0.1±0.8 | 1.4±0.6 | <.001 | 0.6±0.7 | 1.5±0.6 | 1.6±0.5 | <.001 |
| **Number of previous treatment lines, n (%)** | | | | | | | | | | | | |
| 0 | 1815 (37.4) | 1814 (55.2) | 1 (0.1) | <.001 | 1140 (56.7) | 580 (33.4) | 95 (8.6) | <.001 | 1773 (51.8) | 39 (3.5) | 3 (1.0) | <.001 |
| 1 | 1825 (37.6) | 1243 (37.8) | 582 (37.3) |  | 686 (34.1) | 641 (36.9) | 498 (45.2) |  | 1205 (35.2) | 497 (44.3) | 123 (41.0) |  |
| 2 | 1208 (24.9) | 230 (7.0) | 978 (62.7) |  | 185 (9.2) | 515 (29.7) | 508 (46.1) |  | 447 (13.1) | 587 (52.3) | 174 (58.0) |  |
| **Pre-treatment and parenteral nutrition, n (%)** | | | | | | | | | | | | |
| 0 | 318 (90.9) | 235 (92.2) | 83 (87.4) | .267 | 85 (100.0) | 91 (75.8) | 142 (97.9) | <.001 | 200 (98.0) | 84 (81.6) | 34 (79.1) | <.001 |
| 1 | 31 (8.9) | 19 (7.5) | 12 (12.6) |  | - | 28 (23.3) | 3 (2.1) |  | 4 (2.0) | 19 (18.4) | 8 (18.6) |  |
| 2 | 1 (0.3) | 1 (0.4) | - |  | - | 1 (0.8) | - |  | - | - | 1 (2.3) |  |
| **Laboratory values,** **mean (SD)** | | | | | | | | | | | | |
| eGFR^a^ | 60.8 (28.5) | 60.0 (28.6) | 62.3 (28.3) | .43 | 75.1 (23.2) | 38.5 (19.7) | 70.7 (27.0) | <.001 | 64.6 (27.5) | 54.3 (28.2) | 50.5 (32.8) | .001 |
| eGFR (MDV specific)^b^ | 58.7 (27.5) | 58.2 (28.0) | 59.9 (26.3) | .46 | 71.9 (24.4) | 40.1 (20.5) | 68.7 (24.6) | <.001 | 61.9 (27.2) | 52.2 (25.9) | 49.6 (30.9) | <.001 |
| Leukocytes/μL ^c^ | 5433.0 (4672.9) | 5720.5 (5444.5) | 4819.2 (2162.6) | .003 | 5178.8 (3070.1) | 5304.8 (2555.9) | 6188.0 (8727.4) | .120 | 5016.3 (2428.2) | 5748.4 (3780.3) | 9191.8 (15814.6) | <.001 |
| Neutrophil (%)^d^ | 58.8 (15.6) | 58.8 (15.8) | 58.9 (15.2) | .93 | 55.5 (15.8) | 62.1 (14.4) | 59.2 (16.2) | <.001 | 56.9 (15.4) | 61.9 (15.2) | 68.4 (13.7) | <.001 |
| **Disease at the treatment initiation, n (%)** | | | | | | | | | | | | |
| Pneumonia | 175 (3.6) | 6 (0.2) | 169 (10.8) | <.001 | 2 (0.1) | 87 (5.0) | 86 (7.8) | <.001 | 27 (0.8) | 90 (8.0) | 58 (19.3) | <.001 |
| Depression | 95 (2.0) | 10 (0.3) | 85 (5.4) | <.001 | 6 (0.3) | 29 (1.7) | 60 (5.4) | <.001 | 11 (0.3) | 51 (4.5) | 33 (11.0) | <.001 |
| Cancer | 772 (19.7) | 344 (13.3) | 428 (32.4) | <.001 | 274 (16.7) | 254 (19.9) | 244 (24.5) | <.001 | 340 (12.5) | 318 (34.0) | 114 (45.1) | <.001 |
| GERD | 661 (13.6) | 74 (2.3) | 587 (37.6) | <.001 | 33 (1.6) | 206 (11.9) | 422 (38.3) | <.001 | 97 (2.8) | 381 (33.9) | 183 (61.0) | <.001 |
| Other bowel dysfunction | 599 (12.4) | 76 (2.3) | 523 (33.5) | <.001 | 32 (1.6) | 192 (11.1) | 375 (34.1) | <.001 | 79 (2.3) | 340 (30.3) | 180 (60.0) | <.001 |
| Primary HTN | 377 (7.8) | 51 (1.6) | 326 (20.9) | <.001 | 29 (1.4) | 124 (7.1) | 224 (20.3) | <.001 | 61 (1.8) | 207 (18.4) | 109 (36.3) | <.001 |
| Pain | 249 (5.1) | 21 (0.6) | 228 (14.6) | <.001 | 8 (0.4) | 64 (3.7) | 177 (16.1) | <.001 | 21 (0.6) | 131 (11.7) | 97 (32.3) | <.001 |
| Purine and pyrimidine metabolism disorders | 254 (5.2) | 36 (1.1) | 218 (14.0) | <.001 | 11 (0.5) | 77 (4.4) | 166 (15.1) | <.001 | 23 (0.7) | 146 (13.0) | 85 (28.3) | <.001 |
| T2DM | 185 (3.8) | 23 (0.7) | 162 (10.4) | <.001 | 5 (0.2) | 76 (4.4) | 104 (9.4) | <.001 | 15 (0.4) | 93 (8.3) | 77 (25.7) | <.001 |
| **DoC requirement at MM diagnosis^f^, mean±SD** | 0.6±1.5 | 0.4±1.3 | 0.7±1.7 | .001 | 0.2±0.8 | 1.0±1.9 | 0.7±1.5 | <.001 | 0.3±1.1 | 0.6±1.5 | 1.3±2.1 | <.001 |
| **DoC requirement at diagnosis of MM, n (%)^f^** | | | | | | | | | | | | |
| 0 | 1015 (82.9) | 626 (85.2) | 389 (79.6) | .045 | 433 (93.7) | 193 (70.2) | 389 (79.9) | <.001 | 571 (87.7) | 328 (81.4) | 116 (68.2) | <.001 |
| 1 | 42 (3.4) | 28 (3.8) | 14 (2.9) |  | 8 (1.7) | 15 (5.5) | 19 (3.9) |  | 26 (4.0) | 12 (3.0) | 4 (2.4) |  |
| 2 | 43 (3.5) | 22 (3.0) | 21 (4.3) |  | 7 (1.5) | 14 (5.1) | 22 (4.5) |  | 15 (2.3) | 19 (4.7) | 9 (5.3) |  |
| 3 | 31 (2.5) | 17 (2.3) | 14 (2.9) |  | 3 (0.6) | 14 (5.1) | 14 (2.9) |  | 11 (1.7) | 11 (2.7) | 9 (5.3) |  |
| ≥4 | 93 (7.7) | 42 (5.7) | 51 (10.3) |  | 11 (2.5) | 39 (14.1) | 43 (8.8) |  | 28 (4.3) | 33 (8.8) | 32 (18.8) |  |

Missing values from total samples: ^a^4428, ^b^4217, ^c^4221, ^d^4420, ^e^4254, ^f^3624.

CCI, Charlson Comorbidity Index; DoC, degree of care; DoT, duration of treatment; eGFR, estimated glomerular filtration rate; GERD, gastro esophageal reflux disease; HTN, hypertension; LoT, line of treatment; MDV, Medical Data Vision; MM, multiple myeloma; T2DM, type 2 diabetes mellitus.
