# Supplementary figures and images for: Assessment of Predictive Factors That Shorten Duration of Treatment in Patients With Multiple Myeloma Using AI: Real-World Longitudinal Study Using Data From Medical Data Vision Claims Database
Source: JMIR Cancer. 2026 Feb 19;12:e75586. doi: 10.2196/75586 (PMC12963979; doi:10.2196/75586)

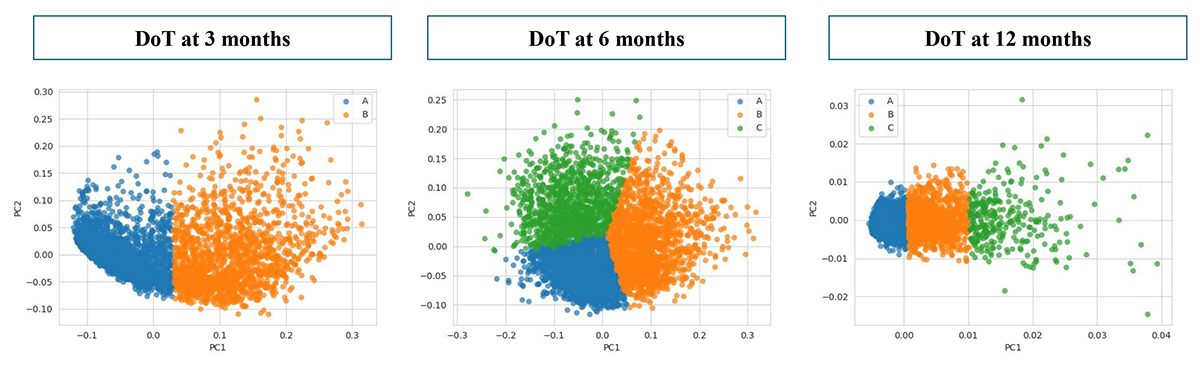

Supplement: Multimedia Appendix 3 [file cancer_v12i1e75586_app3.png]
